# Thermal plasticity of wing size and wing spot size in *Drosophila guttifera*

**DOI:** 10.1007/s00427-023-00705-x

**Published:** 2023-06-19

**Authors:** Yuichi Fukutomi, Aya Takahashi, Shigeyuki Koshikawa

**Affiliations:** 1 Department of Evolution and Ecology, University of California, Davis, One Shields Ave, Davis, CA 95616 USA; 2 Department of Biological Sciences, Tokyo Metropolitan University, 1-1 Minamiosawa, Hachioji, 192-0397 Japan; 3 Research Center for Genomics and Bioinformatics, Tokyo Metropolitan University, 1-1 Minamiosawa, Hachioji, 192-0397 Japan; 4 Graduate School of Environmental Science, Hokkaido University, N10W5, Kita-Ku, Sapporo, Hokkaido 060-0810 Japan; 5 Faculty of Environmental Earth Science, Hokkaido University, N10W5, Kita-Ku, Sapporo, Hokkaido 060-0810 Japan

**Keywords:** *Drosophila guttifera*, Thermal plasticity, Color pattern formation, Morphogen, Image binarization

## Abstract

Thermal plasticity of melanin pigmentation patterns in *Drosophila* species has been studied as a model to investigate developmental mechanisms of phenotypic plasticity. The developmental process of melanin pigmentation patterns on wings of *Drosophila* is divided into two parts, prepattern specification during the pupal period and wing vein-dependent transportation of melanin precursors after eclosion. Which part can be affected by thermal changes? To address this question, we used polka-dotted melanin spots on wings of *Drosophila guttifera*, whose spot areas are specified by *wingless* morphogen. In this research, we reared *D. guttifera* at different temperatures to test whether wing spots show thermal plasticity. We found that wing size becomes larger at lower temperature and that different spots have different reaction norms. Furthermore, we changed the rearing temperature in the middle of the pupal period and found that the most sensitive developmental periods for wing size and spot size are different. The results suggest that the size control mechanisms for the thermal plasticity of wing size and spot size are independent. We also found that the most sensitive stage for spot size was part of the pupal period including stages at which *wingless* is expressed in the polka-dotted pattern. Therefore, it is suggested that temperature change might affect the prepattern specification process and might not affect transportation through wing veins.

**Supplementary Information:**

The online version contains supplementary material available at 10.1007/s00427-023-00705-x.

## Introduction

Organisms show changes in morphology, physiology, or behavior when they face different environmental conditions and these phenomena are called phenotypic plasticity (West-Eberhard 1989). Phenotypic plasticity of animal color patterns can be observed in various taxa, for example, thermal plasticity of pigmentation patterns on the wings of butterflies and the abdomen of fruit flies, changes of feather coloration on birds in response to diet, and rapid camouflage of flatfishes to match their backgrounds (Nijhout 1984; David et al. 1990; Ramachandran et al. 1996; Brakefield et al. 1998; Price 2006; Lafuente et al. 2021). Investigation of developmental mechanisms for color pattern formation has contributed greatly to the understanding of molecular mechanisms for phenotypic plasticity of animal color patterns (Tschirren et al. 2003; Gibert et al. 2007; De Castro et al. 2018; van der Burg et al. 2020).

To understand mechanisms for color pattern formation and phenotypic plasticity of color patterns, melanin pigmentation patterns on the abdomen of *Drosophila melanogaster* have been extensively studied. *D. melanogaster* has sexual dimorphic pigmentation patterns on the abdomen. Males have specific dark pigmentation at the posterior part of the abdomen. The region of male-specific pigmentation is specified by transcription factors, *Abdominal-B* (*Abd-B*) and *bric à brac* (*bab*) gene, during the pupal period (Kopp et al. 2000). Female flies have a dark stripe on each abdominal segment and the proportion of the pigmented area which covers the posterior segments drastically increases when the rearing temperature becomes lower (David et al. 1990). For thermal plasticity of abdominal pigmentation of females, *Abd-B* and *bab*, genes responsible for specification of the pigmented region in males, were shown to have contributions (Gibert et al. 2007; De Castro et al. 2018).

*Drosophila* species have melanin pigmentation patterns also on wings (Edwards et al. 2007; Werner et al. 2018; Koshikawa 2020). Wing pigmentation of *Drosophila* has a unique developmental process. Prepattern is specified by transcription factors or signaling genes during late pupal stages, and the transportation of melanin precursors through wing veins, unique structures on wings, promotes pigmentation after eclosion (True et al. 1999). The latter part of the process gives substantial contributions to formation of wing pigmentation. When a wing vein is surgically cut, wing pigmentation is not fully completed in *D. biarmipes* and the shape of a pigmented area is changed in *D. guttifera* (True et al. 1999; Fukutomi et al. 2017). Whether a wing pigmentation pattern exhibits thermal plasticity or not was studied in male-specific spots on the wings of *D. suzukii* and the spot size divided by wing size showed thermal robustness (Varón-González et al. 2020). If wing pigmentation patterns of other *Drosophila* species show thermal plasticity, it would be possible to test whether prepattern specification or transportation of materials is affected by temperature changes.

*Drosophila guttifera* has a polka-dotted melanin pigmentation pattern on the wing. The color pattern of this species has been used in the context of multiple research fields (Fukutomi and Koshikawa 2021; Niida and Koshikawa 2021). In this species, melanin spots can be observed around campaniform sensilla (Lees 1942) and *wingless* gene is expressed at the campaniform sensilla during the pupal period (Werner et al. 2010; Koshikawa et al. 2015; Koseki et al. 2021). Wingless is an inducer of wing pigmentation and is assumed to specify the area of wing spots by diffusion from campaniform sensilla (Werner et al. 2010). In this study, we reared *D. guttifera* at different temperatures, and measured wing size and spot size. We found that wing size shows thermal plasticity and that different spots have different reaction norms of spot size. We also tested which part of the developmental process contributes to the plasticity. To test whether the prepattern specification process or transportation of materials is important to thermal plasticity in *D. guttifera*, we changed the rearing temperature. From the results, we found that part of the pupal period including stages at which *wingless* is expressed in the polka-dotted pattern is the most sensitive period for thermal plasticity.

## Materials and methods

### Rearing flies and preparing samples

A fly stock used in this study was a wild-type strain of *D. guttifera* (stock no. 15130–1971.10) from *Drosophila* species stock center at the University of California, San Diego. Flies were reared with malt food (containing 50 g cornmeal, 50 g malt, 50 g sugar, 40 g yeast, and 5 g agar in 1 L of water). For experiments, 10 adult male flies and 10 adult female flies were crossed in one vial. Adult flies for crosses were removed four days later. When progenies became adults, they were collected within 24 h after eclosion. To analyze the phenotypic plasticity of wing spots, we reared flies under 18 °C, 21 °C, 25 °C, and 28 °C. As food tends to be dried up at higher temperatures, we placed all fly vials in plastic bags in which moist tissue paper is placed.

For experiments to change the temperature during pupal stages, we picked up pupae at stage P4 (i) (Fukutomi et al. 2017, 2018) and moved them onto moist tissue paper in a Petri dish. P4 (i) is the distinguishable stage without dissection and it is right before the stage when the expression of *wingless* starts in the polka-dotted pattern (Werner et al. 2010; Fukutomi et al. 2017). Flies were reared under the following three conditions.

- “Condition 1”:
  Until P4 (i), flies were reared at 18 °C. From P4 (i), they were reared at 25 °C.
- “Condition 2”:
  Until P4 (i), flies were reared at 25 °C. From P4(i) to P14-15 (stages of pupae), they were reared at 18 °C. At P14-P15, they were moved back to an incubator at 25 °C.
- “Condition 3”:
  Until P14-15, flies were reared at 25 °C. From P14-15, they were reared at 18 °C.

Six to seven days after eclosion, flies were anesthetized with CO_2_. Right wings were dissected and mounted. For mounting solution, a mixture of Hoyer’s solution and lactic acid (1:1 ratio) was used. Photo images (RGB) were taken with a digital camera (DP73, OLYMPUS) connected to a microscope (SZX16, OLYMPUS) by a C-mount adapter (U-TV0.5XC-3, OLYMPUS). The exposure time, aperture (F-number), and ISO were set to 40 ms, 0.075, and 200, respectively. For illumination, reflected light was used (SZX2-RHS, OLYMPUS). For the backgrounds of photo images, the reference greyscale (Brightness = 128, ColorChecker, Xrite) was used.

### Measuring and analyzing wing size and spot size

At first, the brightness of the backgrounds of all photo images was adjusted with ImageJ software (Schneider et al. 2012). The upper right region of each photo image was selected by a rectangle (400 × 300 pixels) and the background was converted by “Window/Level…” function so that the brightness of the selected region became 128.

To estimate the wing size of insects including *Drosophila*, centroid size is frequently used (Debat et al. 2003; Kölliker-Ott et al. 2003; Abbott et al. 2010; Dellicour et al. 2017). Therefore, providing the centroid size data of wings avails intra- and inter-specific comparative analyses (Debat et al. 2003; Gidaszewski et al. 2009). The centroid of a wing is defined as the point which minimizes the sum of squared distances between the point and each landmark on the wing. The centroid size is the square root of the sum of squared distances. In this study, we calculated the centroid size for each wing of *D. guttifera* by the following procedures. The intersection points of veins (Fig. S1a) were used as landmarks. When the landmarks are determined, the coordinates of landmarks and the centroid in each photo image were provided by ImageJ. The centroid size was calculated from those coordinates.

For measuring the spot size, the photo images were binarized with ImageJ. We converted photo images to 8-bit images and selected the polygon indicated in Fig. S1a again. Using “Threshold…” function, 8-bit images of wings were binarized and wing spots became black regions (Fig. S1b). Binarization process was automatically done by Otsu’s method in ImageJ. In Otsu’s method, the histogram of pixel values is divided into two classes by a threshold. The threshold that minimize the intra-class variance and maximize inter-class variance is adopted for binarization (Otsu 1979). By selecting a black region in the binarized images, the area of a wing spot was calculated. The areas of two spots around campaniform sensilla, “Proximal” and “Middle” (Fig. S1b), were used for analysis. To adjust the spot size with wing size, the spot size was divided by the area of the selected polygon (Fig. S1a) instead of centroid size. This is because units of spot size (area, μm^2^) and centroid size (length, mm) are different. We used the polygon area instead of obtaining the whole wing area by tracing the wing boundary, because the reproducibility is higher by using the predetermined landmarks.

Statistical analyses were conducted with R version 4.2.1 (R Core Team 2022). For analyses of wing size and spot size (non-adjusted), one-way ANOVA and Tukey’s honest significant differences (HSD) test were performed. To analyze spot size (adjusted with wing size) and the ratio of “Proximal” spot size to “Middle” spot size, we performed Kruskal–Wallis rank sum test and pairwise comparisons using Wilcoxon rank sum test. We used Bonferroni correction for adjustment of *p*-values in pairwise comparisons using Wilcoxon rank sum test. To grasp the effect of temperatures (18 °C to 28 °C) and conditions (Condition 1 to 3) to spot size in more detail, we regressed log_10_ (spot size) against log_10_ (polygon area), and performed analyses of covariance (ANCOVA). For the statistical validation, we checked the homogeneity of the regression slopes (no significant interaction between temperature and the log of polygon area) by two-way ANOVA. After we confirmed the homogeneity of the regression slopes, we performed ANCOVA with temperature or condition as a fixed factor, and log_10_ (polygon area) as a covariate. For post hoc analysis, we conducted pairwise comparisons of estimated marginal means with Bonferroni correction. In post hoc analysis, we used log_10_ (polygon area) as a covariate. All graphs were produced with ggplot2 (Wickham 2009).

## Results

### Wing size and spot size under different temperatures

**Fig. 1:**
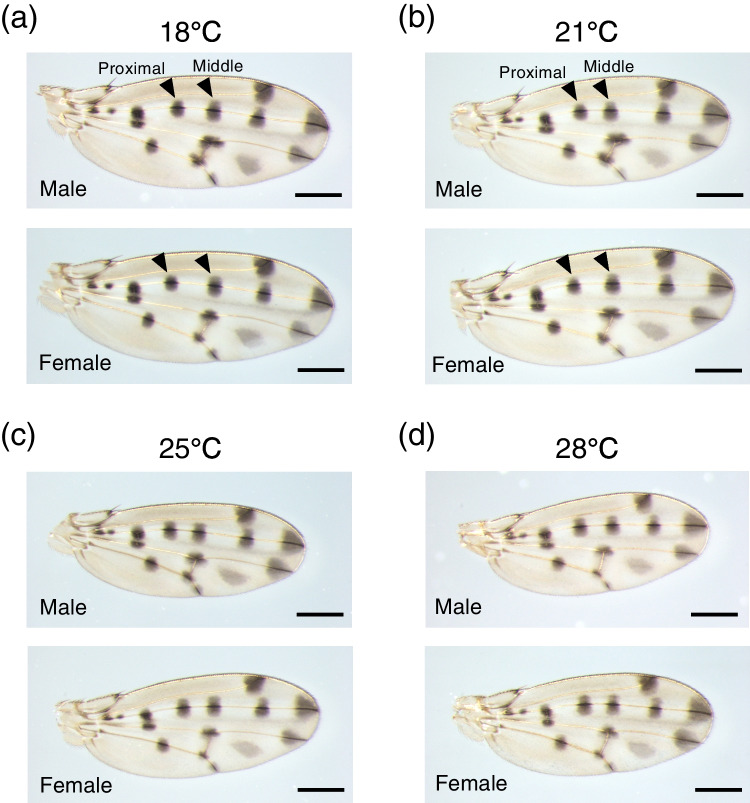
Wings from male and female flies reared at 18 ℃, 21 ℃, 25 ℃, and 28 ℃. **a**: Wings from flies at 18 ℃. The left black arrowheads indicate “Proximal” spots and the right black arrowheads indicate “Middle” spots. **b**: Wings from flies at 21 ℃. **c**: Wings from flies at 25 ℃. **d**: Wings from flies at 28 ℃. To make pictures shown here, the brightness of pictures in Fig. S2 was increased with ImageJ. Scale bars indicate 400 μm

**Fig. 2:**
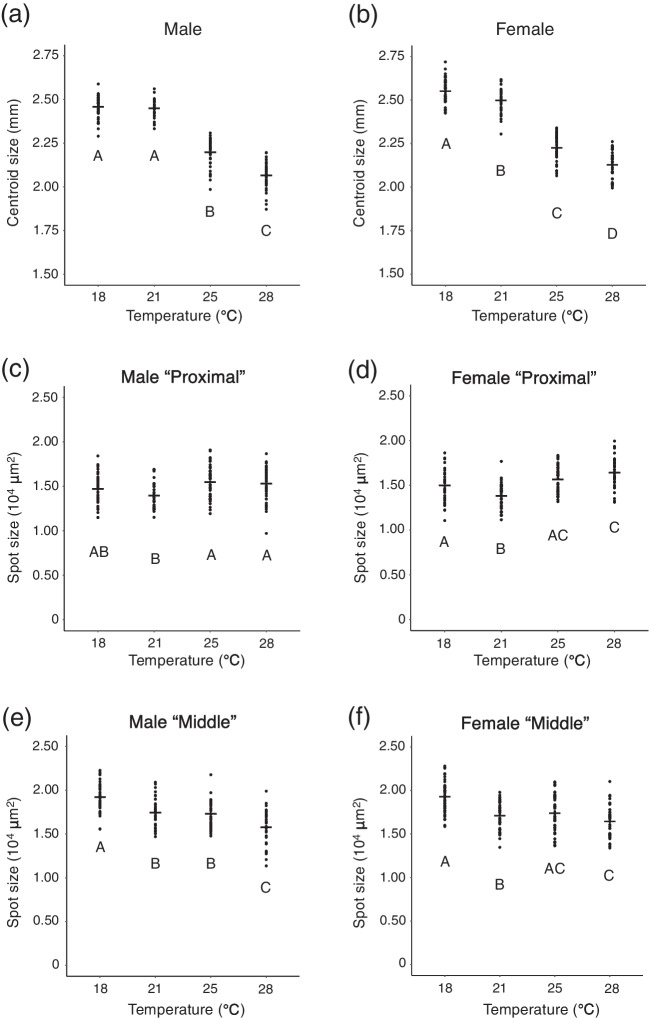
Centroid size of wings and the spot size from flies reared at 18 ℃, 21 ℃, 25 ℃, and 28 ℃. **a**: Centroid size of wings from male flies. **b**: Centroid size of wings from female flies. **c**: Size of “Proximal” spots on wings from male flies. **d**: Size of “Proximal” spots on wings from female flies. **e**: Size of “Middle” spots on wings from male flies. **f**: Size of “Middle” spots on wings from female flies. In all categories, there were significant differences between temperatures (*p* < 0.001, one-way ANOVA, degree of freedom = 3, *F* = 383.9 in **a**, 331.1 in **b**, 6.546 in **c**, 20.83 in **d**, 31.25 in **e**, 18.6 in **f**). Different letters indicate significant differences (*p* < 0.05, Tukey’s HSD test). Black bars indicate mean values

When flies were reared under four different temperatures, wings appeared to be larger at lower temperatures (Fig. 1, S2). We calculated the centroid size and found that it became smaller as the rearing temperature was increased (Fig. 2a, b). That tendency was observed both in males and in females with one exception of a nonsignificant difference between the centroid size at 18 °C and 21 °C in males (Fig. 2a).

We measured the spot size and found that there was no clear tendency correlated with temperature although significant differences between different temperatures were detected in all categories by one-way ANOVA (Fig. 2c-f). The size of “Proximal” spots was almost stable in male flies, as no significant difference was detected between the spot size at 18 °C, 25 °C, and 28 °C by Tukey’s HSD test (Fig. 2c). The size of “Proximal” spots in female flies was not as stable as that in male flies (Fig. 2d). It is possible to interpret that the size of “Middle” spots in males became smaller when the temperature got higher (Fig. 2e), but it is not possible to interpret the data on the size of “Middle” spots in females in the same way (Fig. 2f).

**Fig. 3:**
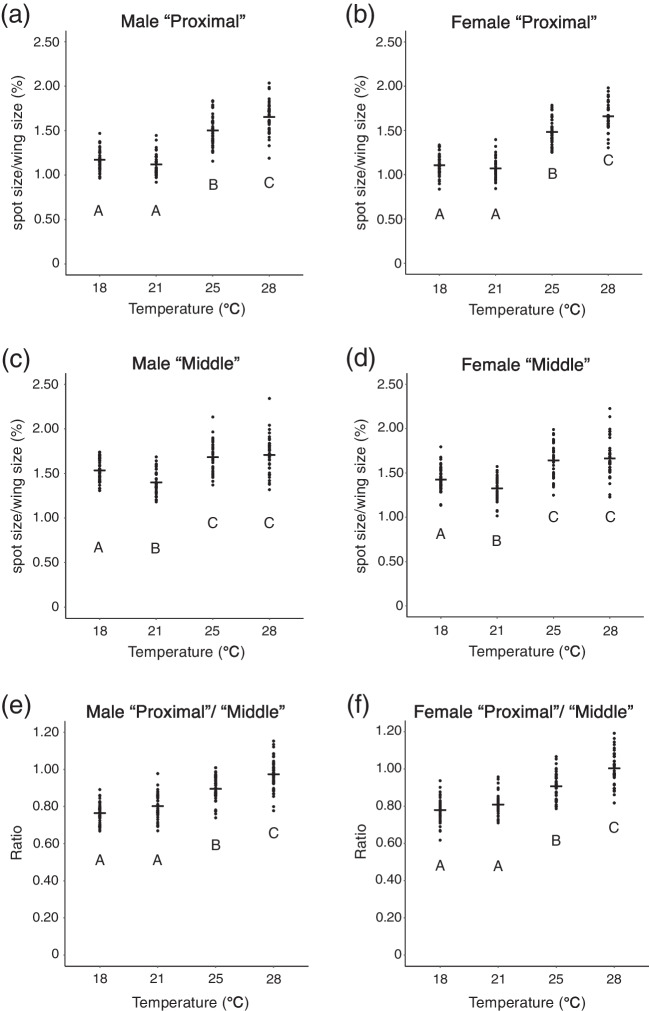
The spot size adjusted with wing size and the ratio of “Proximal” spot size to “Middle” spot size. Flies were reared at 18 ℃, 21 ℃, 25 ℃, and 28 ℃. **a**: Size of “Proximal” spots adjusted with wing size from male flies. **b**: Size of “Proximal” spots adjusted with wing size from female flies. **c**: Size of “Middle” spots adjusted with wing size from male flies. **d**: Size of “Middle” spots adjusted with wing size from female flies. **e**: The ratio in male flies. **f**: The ratio in female flies. In all categories, there were significant differences between temperatures (*p* < 10^–13^, Kruskal–Wallis rank sum test, degree of freedom = 3, *χ*^*2*^ = 120.35 in **a**, 127.11 in **b**, 65.207 in **c**, 77.059 in **d**, 101.22 in **e**, 102.96 in **f**). Different letters indicate significant differences (*p* < 0.05, Wilcoxon rank sum test with Bonferroni correction). Black bars indicate mean values

As centroid size of wings and the area of the polygon mentioned above are highly correlated (Fig. S3), we divided the spot size by the area of the polygon to adjust the spot size by the wing size. After the adjustment, we found that the ratio of the spot size to the wing size was higher at higher temperatures (Fig. 3a-d). Both in males and females, a significant difference between the ratio of “Proximal” size to wing size at 25 °C and the ratio at 28 °C was observed by Wilcoxon rank sum test with Bonferroni correction (Fig. 3a, b). For “Middle” spots, a significant difference between the ratio at 18 °C and that at 21 °C was detected by Wilcoxon rank sum test with Bonferroni correction (Fig. 3c, d).

As a conspicuous result, we noticed that “Proximal” spot was smaller than “Middle” spot at 18 °C and 21 °C (Fig. 1a, b). When we analyzed the ratio of “Proximal” size to “Middle” size, we found that the ratio became higher when the rearing temperature became higher (Fig. 3e, f). Other than the comparison between ratios at 18 °C and those at 21 °C, significant differences were detected by Wilcoxon rank sum test with Bonferroni correction both in males and females (Fig. 3e, f).

**Fig. 4:**
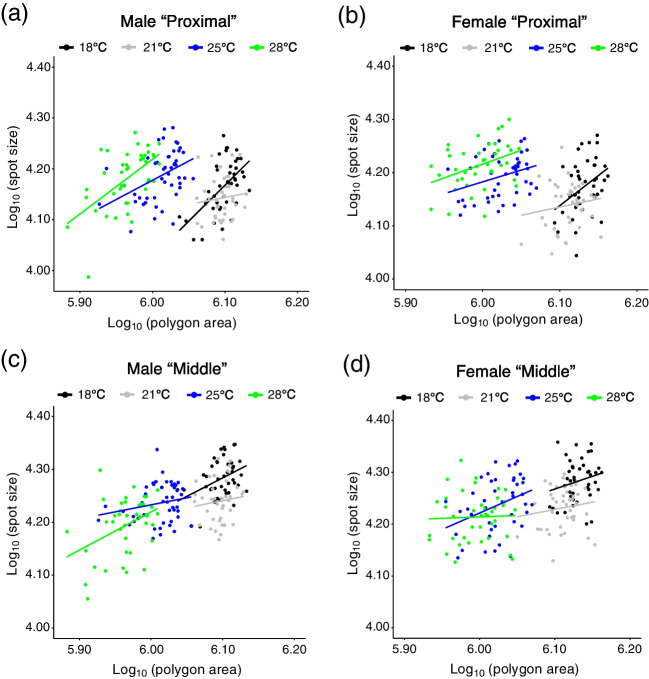
Log–log plot of polygon area (μm^2^) and spot size (μm^2^). The spot size is from flies reared at 18 ℃, 21 ℃, 25 ℃, and 28 ℃. **a**: “Proximal” spots on wings from male flies. **b**: “Proximal” spots on wings from female flies. **c**: “Middle” spots on wings from male flies. **d**: “Middle” spots on wings from female flies

When log_10_ (spot size) is regressed against log_10_ (polygon size), we found that the correlation between log_10_ (spot size) and log_10_ (polygon size) was the strongest in samples for male “Proximal” spots (Fig. 4, Table S1). As results for testing whether there is an interaction between log_10_ (polygon size) and temperature (testing whether regression lines have different slopes or not), no significance was detected in any categories by two-way ANOVA (Table S2). By ANCOVA, it was shown that the effect of temperature was significant in all categories (Table 1). The results of the post hoc analyses are written in Table 2. For “Proximal” spots in males and females, other than the comparison between 18 °C and 21 °C, significant differences were detected. For “Middle” spots in males, significant differences were detected in the comparison between 18 °C and 21 °C, and that between 21 °C and 25 °C. In female “Middle” spots, the significant difference was detected only in the comparison between 18 °C and 21 °C.

**Table 1:** The results of ANCOVA analysis with “Temperature” as a fixed factor and “Log_10_ (polygon area)” as a covariate. Sum Sq: sum of squares, Df: degrees of freedom, ***: *p* ≦ 0.001, **: *p* ≦ 0.01, *: *p* < 0.05

Effect	Sum Sq	Df	*F* value	*p* value
Male “Proximal” (Fig. 4a)				
Log_10_ (polygon area)	0.089	1	43.435	***
Temperature	0.136	3	22.078	***
Residuals	0.340	165		
Female “Proximal” (Fig. 4b)				
Log_10_ (polygon area)	0.030	1	16.277	***
Temperature	0.116	3	21.310	***
Residuals	0.296	163		
Male “Middle” (Fig. 4c)				
Log_10_ (polygon area)	0.026	1	15.915	***
Temperature	0.045	3	8.939	***
Residuals	0.274	165		
Female “Middle” (Fig. 4d)				
Log_10_ (polygon area)	0.013	1	6.246	*
Temperature	0.047	3	7.804	***
Residuals	0.329	163		
Male “Proximal” (The rearing temperature is changed in the pupal period, Fig. 7a)				
Log_10_ (polygon area)	0.056	1	29.631	***
Condition	0.156	2	41.446	***
Residuals	0.203	108		
Female “Proximal” (The rearing temperature is changed in the pupal period, Fig. 7b)				
Log_10_ (polygon area)	0.017	1	10.090	**
Condition	0.271	2	78.447	***
Residuals	0.186	108		
Male “Middle” (The rearing temperature is changed in the pupal period, Fig. 7c)				
Log_10_ (polygon area)	0.016	1	9.798	**
Condition	0.032	2	9.843	***
Residuals	0.178	108		
Female “Middle” (The rearing temperature is changed in the pupal period, Fig. 7d)				
Log_10_ (polygon area)	0.018	1	8.267	**
Condition	0.054	2	12.477	***
Residuals	0.234	108

**Table 2:** The results of post hoc pairwise comparisons of estimated marginal means with Bonferroni correction. ***: *p* ≦ 0.001, **: *p* ≦ 0.01, *: *p* < 0.05, NS: Not Significant

Comparison	Significance
Male “Proximal” (Fig. 4a)	
18 ℃ and 21 ℃	NS
18 ℃ and 25 ℃	***
18 ℃ and 28 ℃	***
21 ℃ and 25 ℃	***
21 ℃ and 28 ℃	***
25 ℃ and 28 ℃	**
Female “Proximal” (Fig. 4b)	
18 ℃ and 21 ℃	NS
18 ℃ and 25 ℃	***
18 ℃ and 28 ℃	***
21 ℃ and 25 ℃	***
21 ℃ and 28 ℃	***
25 ℃ and 28 ℃	**
Male “Middle” (Fig. 4c)	
18 ℃ and 21 ℃	***
18 ℃ and 25 ℃	NS
18 ℃ and 28 ℃	NS
21 ℃ and 25 ℃	*
21 ℃ and 28 ℃	NS
25 ℃ and 28 ℃	NS
Female “Middle” (Fig. 4d)	
18 ℃ and 21 ℃	***
18 ℃ and 25 ℃	NS
18 ℃ and 28 ℃	NS
21 ℃ and 25 ℃	NS
21 ℃ and 28 ℃	NS
25 ℃ and 28 ℃	NS
Male “Proximal” (The rearing temperature is changed in the pupal period, Fig. 7a)	
Condition 1 and Condition 2	***
Condition 1 and Condition 3	NS
Condition 2 and Condition 3	***
Female “Proximal” (The rearing temperature is changed in the pupal period, Fig. 7b)	
Condition 1 and Condition 2	***
Condition 1 and Condition 3	NS
Condition 2 and Condition 3	***
Male “Middle” (The rearing temperature is changed in the pupal period, Fig. 7c)	
Condition 1 and Condition 2	***
Condition 1 and Condition 3	NS
Condition 2 and Condition 3	*
Female “Middle” (The rearing temperature is changed in the pupal period, Fig. 7d)	
Condition 1 and Condition 2	**
Condition 1 and Condition 3	NS
Condition 2 and Condition 3	***

### Change of wing size and spot size when the rearing temperature is changed

**Fig. 5:**
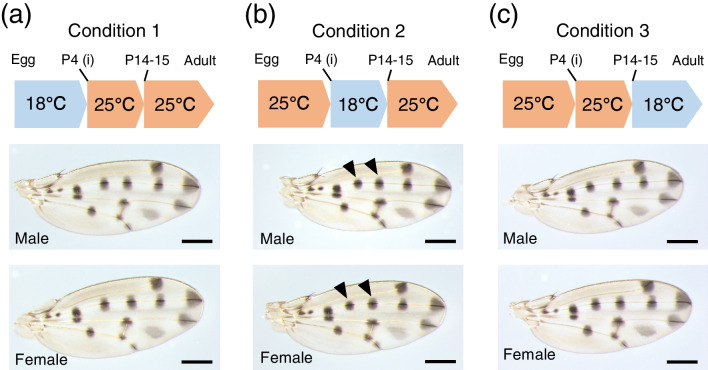
Wings from male and female flies whose rearing temperatures were changed during the pupal period. **a**: Wings from male and female flies reared under “Condition 1”. Until P4 (i), flies were reared at 18 ℃. From P4 (i), they were reared at 25 ℃. **b**: Wings from male and female flies reared under “Condition 2”. Until P4 (i), flies were reared at 25 ℃. From P4(i) to P14-15, they were reared at 18 ℃. From P14-P15, they were reared at 25 ℃. The left black arrowheads indicate “Proximal” spots and the right black arrowheads indicate “Middle” spots. **c**: Wings from male and female flies reared under “Condition 3”. Until P14-15, flies were reared at 25 ℃. From P14-15, they were reared at 18 ℃. For all pictures, the brightness of the background was increased with ImageJ. Scale bars indicate 400 μm

**Fig. 6:**
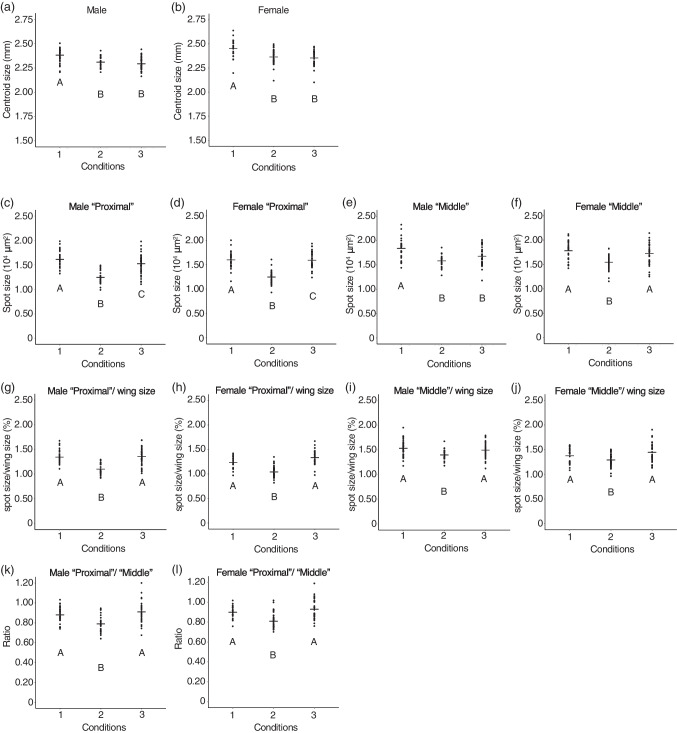
The results form analyses of flies reared under “Condition 1”, “Condition 2”, and “Condition 3”. Centroid size of wings from flies, the spot size, the spot size adjusted with wing size, and the ratio of “Proximal” spot size to “Middle” spot size are indicated. **a**: Centroid size of wings from male flies. **b**: Centroid size of wings from female flies. **c**: Size of “Proximal” spots on wings from male flies. **d**: Size of “Proximal” spots on wings from female flies. **e**: Size of “Middle” spots on wings from male flies. **f**: Size of “Middle” spots on wings from female flies. **g**: Size of “Proximal” spots adjusted with wing size from male flies. **h**: Size of “Proximal” spots adjusted with wing size from female flies. **i**: Size of “Middle” spots adjusted with wing size from male flies. **j**: Size of “Middle” spots adjusted with wing size from female flies. **k**: The ratio in male flies. **l**: The ratio in female flies. From **a** to **f**, there were significant differences between temperatures (*p* < 10^–5^, one-way ANOVA, degree of freedom = 2, *F* = 24.64 in **a**, 15.43 in **b**, 39.75 in **c**, 78.34 in **d**, 20.07 in **e**, 17.77 in **f**). Different letters indicate significant differences (*p* < 0.05, Tukey’s HSD test). From **g** to **l**, there were significant differences between temperatures (*p* < 10^–5^, Kruskal–Wallis rank sum test, degree of freedom = 2, *χ*^*2*^ = 45.315 in **g**, 62.827 in **h**, 15.805 in **i**, 16.804 in **j**, 6.414 in **k**, 42.698 in **l**). Different letters indicate significant differences (*p* < 0.05, Wilcoxon rank sum test with Bonferroni correction). Black bars indicate mean values

By rearing *D. guttifera* under different temperatures, it was shown that the wing size and the spot size of *D. guttifera* exhibit phenotypic plasticity. To investigate which stage is sensitive to temperature, we changed the rearing temperature during the pupal period. The wing size was the largest when flies were reared under “Condition 1” (reared at 18 °C until P4 (i)) (Fig. 5). By Tukey’s HSD test, significant differences were detected between the wing size of the flies reared under “Condition 1” and the other two conditions (Fig. 6a, b). No significant difference was detected between “Condition 2” and “Condition 3” (Fig. 6a, b). The same tendency was observed both in males and females (Fig. 6a, b).

When we measured spot size, we found that the mean spot size of wings from flies reared under “Condition 2” (reared at 18 °C from P4 (i) to P14-15) became the smallest (Fig. 6c-f). Both in males and females, all comparisons of “Proximal” spot size showed significant differences by Tukey’s HSD test (Fig. 6c, d). For “Middle” spot size, a significant difference between the spot size of the flies reared under “Condition 1” and “Condition 3” was detected in males by Tukey’s HSD test (Fig. 6e), but it was not detected in females (Fig. 6f).

When the spot size is adjusted with wing size, the mean spot size of the wings from flies reared under “Condition 2” became the smallest in all categories (Fig. 6g-j). No significant difference between the spot size of flies reared under “Condition 1” and “Condition 3” was detected in any category (Fig. 6g-j).

Under “Condition 2”, “Proximal” spot was smaller than “Middle” spot (Fig. 5b). Analyzing the ratio of “Proximal” size to “Middle” size, we found that the ratio becomes smaller under “Condition 2” both in males and females (Fig. 6k, l). A significant difference between the spot size of the flies reared under “Condition 1” and “Condition 3” was not observed (Fig. 6k, l).

**Fig. 7:**
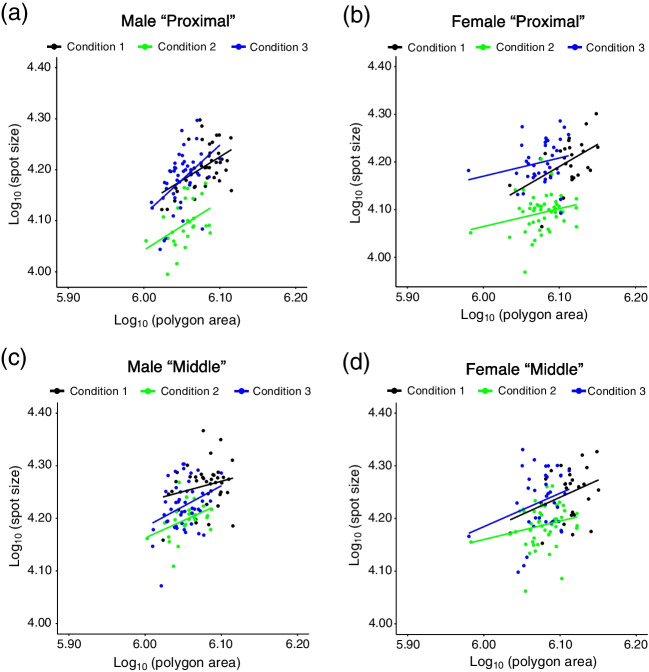
Log–log plot of spot size (μm^2^) and polygon area (μm^2^). The spot size is from flies whose rearing temperatures were changed during the pupal period. **a**: “Proximal” spots on wings from male flies. **b**: “Proximal” spots on wings from female flies. **c**: “Middle” spots on wings from male flies. **d**: “Middle” spots on wings from female flies

When log_10_ (spot size) is regressed against log_10_ (polygon size), we found that the correlation between log_10_ (spot size) and log_10_ (polygon size) was the strongest in male “Proximal” spots (Fig. 7, Table S1). As results for testing whether there is an interaction between log_10_ (polygon size) and conditions (testing whether regression lines have different slopes or the not), no significance was detected in any categories by two-way ANOVA (Table S2). By ANCOVA, it was shown that the effect of conditions was significant in all categories (Table 1). In the results of the post hoc analyses, significant differences between “Condition 1” and “Condition 2”, and between “Condition 2” and “Condition 3” were detected in all categories. No significant difference between “Condition 1” and “Condition 3” was detected in any category (Table 2).

## Discussion

In this study, we found that the wing size of *D. guttifera* shows thermal plasticity and that different spots have different reaction norms. The tendency of thermal plasticity we observed was similar between males and females. Wing size becomes larger when flies are reared at lower temperatures (Figs. 1 and 2a, b) as reported in other *Drosophila* species (Crill et al. 1996; Debat et al. 2003; Gilchrist and Huey 2004; Varón-González et al. 2020). Spot size itself changes between different temperatures (significant differences were observed by one-way ANOVA, Fig. 2c-f), but to grasp the tendency of spot size itself correlated with temperature is difficult from the results. When spot size is adjusted with wing size, the adjusted spot size became larger when the rearing temperature was higher (Fig. 3a-d). This might be because changes in wing size depending on temperature are much more drastic than changes in spot size itself (Fig. 2). Differences in the ratio between the size of “Proximal” spots and the size of “Middle” spots depending on rearing temperature showed that “Proximal” spot and “Middle” spot have different reaction norms (Fig. 3e, f). From ANCOVA results, the difference in response to temperature (different scaling relationships with wing size) between “Proximal” spots and “Middle” spots was elucidated in more detail. The results indicate that temperature regulates “Proximal” spot size independently of the temperature effect on changes in wing size both in males and females. Although the effect of temperature on “Middle” spot size was significant, the results from post hoc analysis suggest that “Middle” spot size mainly changes due to the temperature effect on wing size. Taken together, it is suggested that “Middle” spot size is much more robust to temperature, compared to “Proximal” spot size (Fig. 4, Tables 1 and 2).

The results from experiments in which the rearing temperature is changed during the pupal period suggest that the thermal plasticity of wing size and that of spot size are independently regulated. For wing size, developmental stages before P4 (i) are the most sensitive period to the rearing temperature (Fig. 6a, b). This result is similar to the tendency of wing size plasticity in *D. melanogaster* which shows that earlier developmental stages are more sensitive to the rearing temperature than later stages, such as pupal stages (French et al. 1998). For spot size, the period from P4 (i) to P14-15 is the most sensitive because spot size (absolute size and adjusted size) becomes the smallest and “Proximal” spot is smaller than “Middle” spot when flies are exposed to lower temperatures from P4 (i) to P14-15 (Fig. 6c-j). This is also supported by ANCOVA results. When the wing size was set as a covariate, spot size of flies exposed to 18 °C before P4 (i) and since P14-15 did not show significant difference. The spot size of flies 18 °C from P4 (i) to P14-15 was significantly different from the other two conditions (Fig. 7, Tables 1 and 2). As a conspicuous phenotype, we observed that “Proximal” spot is smaller than “Middle” spot when flies were reared at lower temperatures (Fig. 1a, b). This phenotype was observed only when flies were exposed to lower temperatures from P4 (i) to P14-15 (Figs. 5 and 6k, l). As the most sensitive stages for wing size and those for spot size are different, it is suggested that the different developmental mechanisms produce thermal plasticity for wing size and spot size.

Results obtained in this study also suggest that the transportation of materials through veins after eclosion does not have a considerable effect on the thermal plasticity of wing spots in *D. guttifera*. There was no significant difference between the adjusted spot size of the flies exposed to lower temperatures until P4 (i) and that of flies exposed to lower temperature from P14-15 (Fig. 6g-j). Exposure to 18 °C since pupal stage P14-15 did not produce a difference in spot size between “Proximal” spot and “Middle” spot, the conspicuous phenomenon that can be observed when flies were reared at lower temperatures (Figs. 5 and 6k, l). From ANCOVA results, the spot size of flies exposed to 18 °C before P4 (i) and since P14-15 did not show a significant difference when the wing size was set as a covariate (Fig. 7, Tables 1 and 2). As spot size divided by wing size showed thermal robustness in the male-specific wing spot of *D. suzukii* (Varón-González et al. 2020), changes in rearing temperature do not affect the transportation of materials through veins also in *D. suzukii*. The process of transportation of materials through wing veins might be robust to thermal changes and that tendency might be conserved in *Drosophila* species.

Furthermore, results suggest that the pattern specification process during the pupal period is important to thermal plasticity of wing spots in *D. guttifera*. Expression of *wingless* gene at campaniform sensilla starts at stage P6 (Werner et al. 2010) and the expression at presumptive spot regions can be detected at stage P12 (Fukutomi et al. 2021). After eclosion, epithelial cells at presumptive spot regions, which receive Wingless signaling, disappear (Fukutomi et al. 2017) and it can be considered that specification of pigmented regions by Wingless ends before eclosion. Therefore, stages at which Wingless morphogen specifies the spot regions are included in the period from P4 (i) to P14-15, which is sensitive to the rearing temperature in terms of determining spot size.

One candidate mechanism for producing thermal plasticity and different reaction norms of wing spot size in *D. guttifera* is that the rearing temperature affects the mechanism of determining spot size by Wingless morphogen. The extracellular distribution of morphogens is considered or shown to be plastic to the surrounding temperature (Houchmandzadeh et al. 2002; Eldar et al. 2004; Barkai and Shilo 2009). In the context of color pattern formation in wings, it is considered that changes in the distribution of signaling molecules such as Wingless will alter the outcome patterning (Martin and Reed 2014; Martin and Courtier-Orgogozo 2017; Özsu et al. 2017). In *D. guttifera*, changes in the rearing temperature might affect the distribution of Wingless and produce thermal plasticity in spot size. If the response of Wingless distribution to temperature differs between spots, reaction norms of spot size would differ and produce different scaling relationships with wing size. However, other genetic mechanisms might be responsible for thermal plasticity and differences in reaction norms of spot size.

In conclusion, the size of wing spots around campaniform sensilla of *D. guttifera* shows thermal plasticity and reaction norms are different in different spots. The most sensitive period for the thermal plasticity of spot size includes the pupal stage at which *wingless* is expressed on wings. Our results suggest that the process for specifying the presumptive pigmented areas by Wingless is affected by temperature change, and the transportation of materials through veins after eclosion does not have a considerable effect on the thermal plasticity of wing spots.

### Supplementary Information

Below is the link to the electronic supplementary material.

Supplementary file1 Landmarks and spots on a wing used in this study. a: Landmarks and a polygon used for analyses. Landmarks, intersection points of veins, are indicated as white dots. The polygon was drawn by connecting white dots. The brightness of the background was increased with ImageJ. b: Binarized image of a wing. For convenience, we call the spot indicated with the left soft orange arrowhead as “Proximal”, and call the one indicated with the right soft orange arrowhead as “Middle”. (PDF 929 KB)

Supplementary file2 Wings from male and female flies reared at 18 ℃, 21 ℃, 25 ℃, and 28 ℃. Those pictures were used for measurement of wing size and spot size. In Fig. 1, the brightness of pictures shown here was increased with ImageJ. a: Wings from flies at 18 ℃. The left black arrowheads indicate “Proximal” spots and the right black arrowheads indicate “Middle” spots. b: Wings from flies at 21 ℃. c: Wings from flies at 25 ℃. d: Wings from flies at 28 ℃. Scale bars indicate 400 μm. (PDF 278075 KB)

Supplementary file3 The correlation between centroid size and the area of the polygon. a: Wings of males reared under 18 ℃, 21 ℃, 25 ℃, and 28 ℃. b: Wings of females reared under 18 ℃, 21 ℃, 25 ℃, and 28 ℃. c: Wings of males whose rearing temperatures were changed during the pupal period. d: Wings of males whose rearing temperatures were changed during the pupal period. Grey shadows indicate 95% confidence intervals. (PDF 511 KB)

Supplementary file4 (DOCX 16.7 KB)

## Data Availability

Data obtained in this study are available from figshare at https://figshare.com/articles/dataset/Wing_size_and_wing_spot_size_of_Drosophila_guttifera/21760562
