# Supplementary figures and images for: Thermal plasticity of wing size and wing spot size in Drosophila guttifera
Source: Dev Genes Evol. 2023 Jun 19;233(2):77–89. doi: 10.1007/s00427-023-00705-x (PMC10746645; doi:10.1007/s00427-023-00705-x)

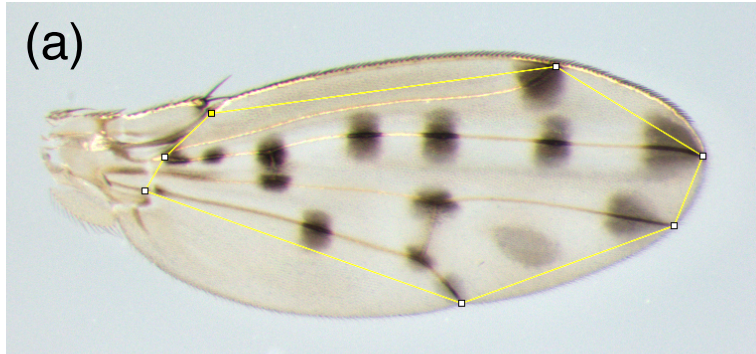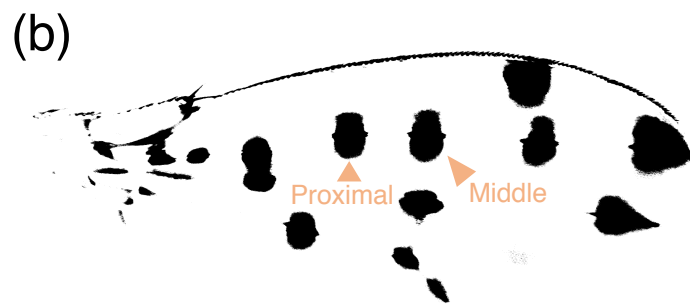

Supplement: Supplementary file 1 — Supplementary file1 Landmarks and spots on a wing used in this study. a: Landmarks and a polygon used for analyses. Landmarks, intersection points of veins, are indicated as white dots. The polygon was drawn by connecting white dots. The brightness of the background was increased with ImageJ. b: Binarized image of a wing. For convenience, we call the spot indicated with the left soft orange arrowhead as “Proximal”, and call the one indicated with the right soft orange arrowhead as “Middle”. (PDF 929 KB) [file 427_2023_705_MOESM1_ESM.pdf]

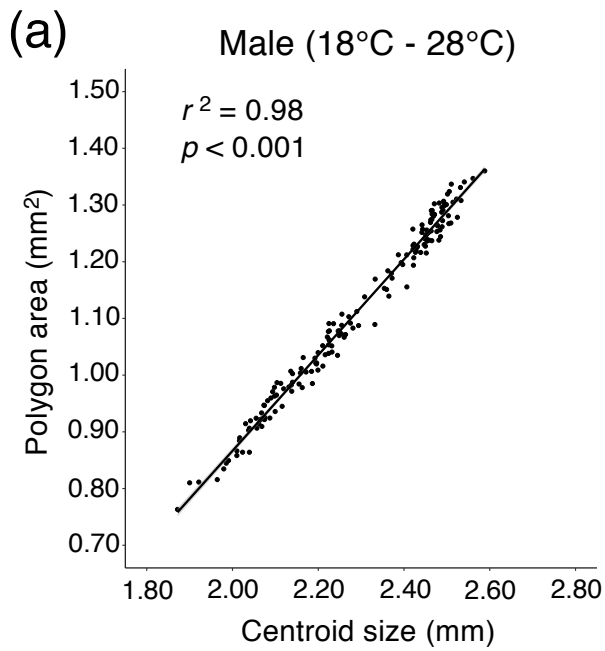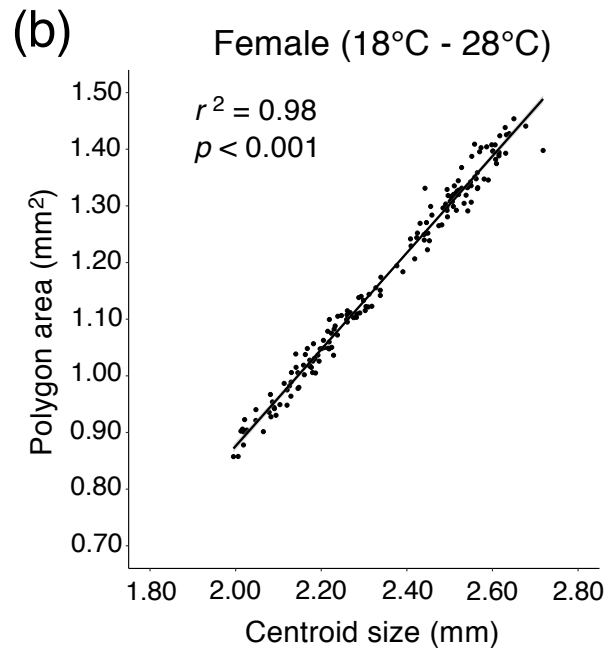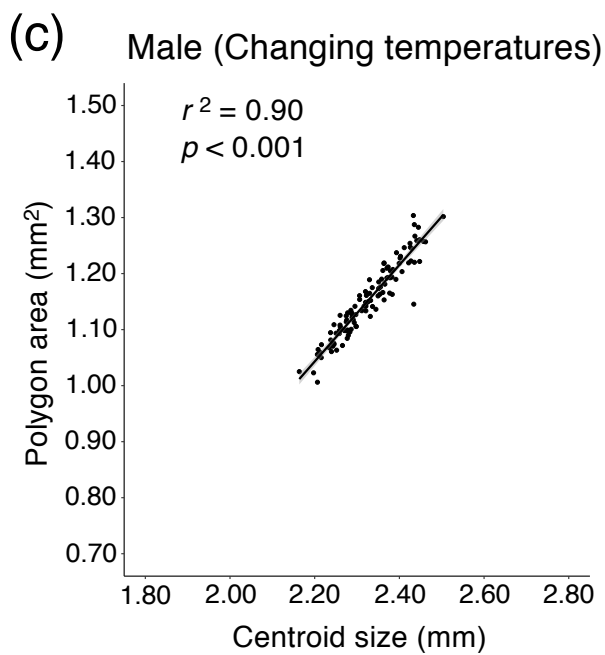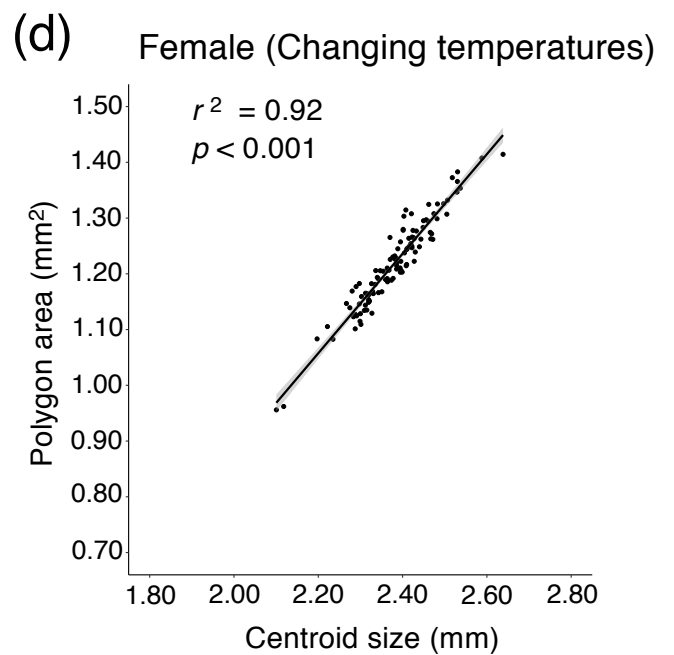

Supplement: Supplementary file 3 — Supplementary file3 The correlation between centroid size and the area of the polygon. a: Wings of males reared under 18 ℃, 21 ℃, 25 ℃, and 28 ℃. b: Wings of females reared under 18 ℃, 21 ℃, 25 ℃, and 28 ℃. c: Wings of males whose rearing temperatures were changed during the pupal period. d: Wings of males whose rearing temperatures were changed during the pupal period. Grey shadows indicate 95% confidence intervals. (PDF 511 KB) [file 427_2023_705_MOESM3_ESM.pdf]
