# Supplementary material for: Thermal plasticity of wing size and wing spot size in Drosophila guttifera
Source: Dev Genes Evol. 2023 Jun 19;233(2):77–89. doi: 10.1007/s00427-023-00705-x (PMC10746645; doi:10.1007/s00427-023-00705-x)
Supplement: Supplementary file 4 — Supplementary file4 (DOCX 16.7 KB) [file 427_2023_705_MOESM4_ESM.docx]

Table S1.

The correlation between log of polygon area and log of spot size. *r*^2^ values and significance of the regression lines in Fig. S4 and S5 are written. ***: *p* ≦ 0.001, **: *p* ≦ 0.01, *: *p* < 0.05, NS: Not Significant.

| Sex | Spot | Temperature/treatment | *r*^2^ | Significance |
| --- | --- | --- | --- | --- |
| Male | Proximal | 18 ℃ (Fig. 4a) | 0.32 | *** |
| Male | Proximal | 21 ℃ (Fig. 4a) | 0.012 | NS |
| Male | Proximal | 25 ℃ (Fig. 4a) | 0.17 | ** |
| Male | Proximal | 28 ℃ (Fig. 4a) | 0.33 | *** |
| Female | Proximal | 18 ℃ (Fig. 4b) | 0.15 | * |
| Female | Proximal | 21 ℃ (Fig. 4b) | 0.021 | NS |
| Female | Proximal | 25 ℃ (Fig. 4b) | 0.10 | * |
| Female | Proximal | 28 ℃ (Fig. 4b) | 0.040 | * |
| Male | Middle | 18 ℃ (Fig. 4c) | 0.15 | * |
| Male | Middle | 21 ℃ (Fig. 4c) | 0.013 | NS |
| Male | Middle | 25 ℃ (Fig. 4c) | 0.040 | NS |
| Male | Middle | 28 ℃ (Fig. 4c) | 0.16 | ** |
| Female | Middle | 18 ℃ (Fig. 4d) | 0.043 | NS |
| Female | Middle | 21 ℃ (Fig. 4d) | 0.020 | NS |
| Female | Middle | 25 ℃ (Fig. 4d) | 0.12 | * |
| Female | Middle | 28 ℃ (Fig. 4d) | 0.0014 | NS |
| Male | Proximal | Condition 1 (Fig. 7a) | 0.24 | ** |
| Male | Proximal | Condition 2 (Fig. 7a) | 0.18 | * |
| Male | Proximal | Condition 3 (Fig. 7a) | 0.23 | *** |
| Female | Proximal | Condition 1 (Fig. 7b) | 0.23 | * |
| Female | Proximal | Condition 2 (Fig. 7b) | 0.058 | NS |
| Female | Proximal | Condition 3 (Fig. 7b) | 0.044 | NS |
| Male | Middle | Condition 1 (Fig. 7c) | 0.047 | NS |
| Male | Middle | Condition 2 (Fig. 7c) | 0.16 | * |
| Male | Middle | Condition 3 (Fig. 7c) | 0.10 | * |
| Female | Middle | Condition 1 (Fig. 7d) | 0.12 | NS |
| Female | Middle | Condition 2 (Fig. 7d) | 0.049 | NS |
| Female | Middle | Condition 3 (Fig. 7d) | 0.074 | NS |

Table S2.

The results of two-way ANOVA. Whether there is a significant interaction between temperature and log of polygon area was tested. DFn: the degree of freedom for the numerator of the *F* ratio, DFd: he degree of freedom for the denominator of the *F* ratio, ***: *p* ≦ 0.001, **: *p* ≦ 0.01, *: *p* < 0.05.

| Effect | DFn | DFd | *F* value | *p* value |
| --- | --- | --- | --- | --- |
| Male “Proximal” (Fig. 4a) | | | | |
| Log_10_ (polygon area) | 1 | 162 | 44.004 | *** |
| Temperature | 3 | 162 | 22.367 | *** |
| Log_10_ (polygon area) : Temperature | 3 | 162 | 1.721 | 0.165 |
| Female “Proximal” (Fig. 4b) | | | | |
| Log_10_ (polygon area) | 1 | 160 | 16.287 | *** |
| Temperature | 3 | 160 | 21.322 | *** |
| Log_10_ (polygon area) : Temperature | 3 | 160 | 1.030 | 0.381 |
| Male “Middle” (Fig. 4c) | | | | |
| Log_10_ (polygon area) | 1 | 162 | 15.914 | *** |
| Temperature | 3 | 162 | 8.939 | *** |
| Log_10_ (polygon area) : Temperature | 3 | 162 | 0.996 | 0.396 |
| Female “Middle” (Fig. 4d) | | | | |
| Log_10_ (polygon area) | 1 | 160 | 6.259 | * |
| Temperature | 3 | 160 | 7.820 | *** |
| Log_10_ (polygon area) : Temperature | 3 | 160 | 1.111 | 0.346 |
| Male “Proximal” (The rearing temperature is changed in the pupal period, Fig. 7a) | | | | |
| Log_10_ (polygon area) | 1 | 106 | 29.378 | *** |
| Temperature | 2 | 106 | 41.093 | *** |
| Log_10_ (polygon area) : Temperature | 2 | 106 | 0.540 | 0.585 |
| Female “Proximal” (The rearing temperature is changed in the pupal period, Fig. 7b) | | | | |
| Log_10_ (polygon area) | 1 | 106 | 10.100 | ** |
| Temperature | 2 | 106 | 78.519 | *** |
| Log_10_ (polygon area) : Temperature | 2 | 106 | 1.049 | 0.354 |
| Male “Middle” (The rearing temperature is changed in the pupal period, Fig. 7c) | | | | |
| Log_10_ (polygon area) | 1 | 106 | 9.699 | ** |
| Temperature | 2 | 106 | 9.743 | *** |
| Log_10_ (polygon area) : Temperature | 2 | 106 | 0.451 | 0.638 |
| Female “Middle” (The rearing temperature is changed in the pupal period, Fig. 7d) | | | | |
| Log_10_ (polygon area) | 1 | 106 | 8.163 | ** |
| Temperature | 2 | 106 | 12.321 | *** |
| Log_10_ (polygon area) : Temperature | 2 | 106 | 0.324 | 0.724 |
