# Supplementary material for: Thermal plasticity of wing size and wing spot size in Drosophila guttifera
Source: Dev Genes Evol. 2023 Jun 19;233(2):77–89. doi: 10.1007/s00427-023-00705-x (PMC10746645; doi:10.1007/s00427-023-00705-x)
Supplement: Supplementary file 2 — Supplementary file2 Wings from male and female flies reared at 18 ℃, 21 ℃, 25 ℃, and 28 ℃. Those pictures were used for measurement of wing size and spot size. In Fig. 1, the brightness of pictures shown here was increased with ImageJ. a: Wings from flies at 18 ℃. The left black arrowheads indicate “Proximal” spots and the right black arrowheads indicate “Middle” spots. b: Wings from flies at 21 ℃. c: Wings from flies at 25 ℃. d: Wings from flies at 28 ℃. Scale bars indicate 400 μm. (PDF 278075 KB) [file 427_2023_705_MOESM2_ESM.pdf]

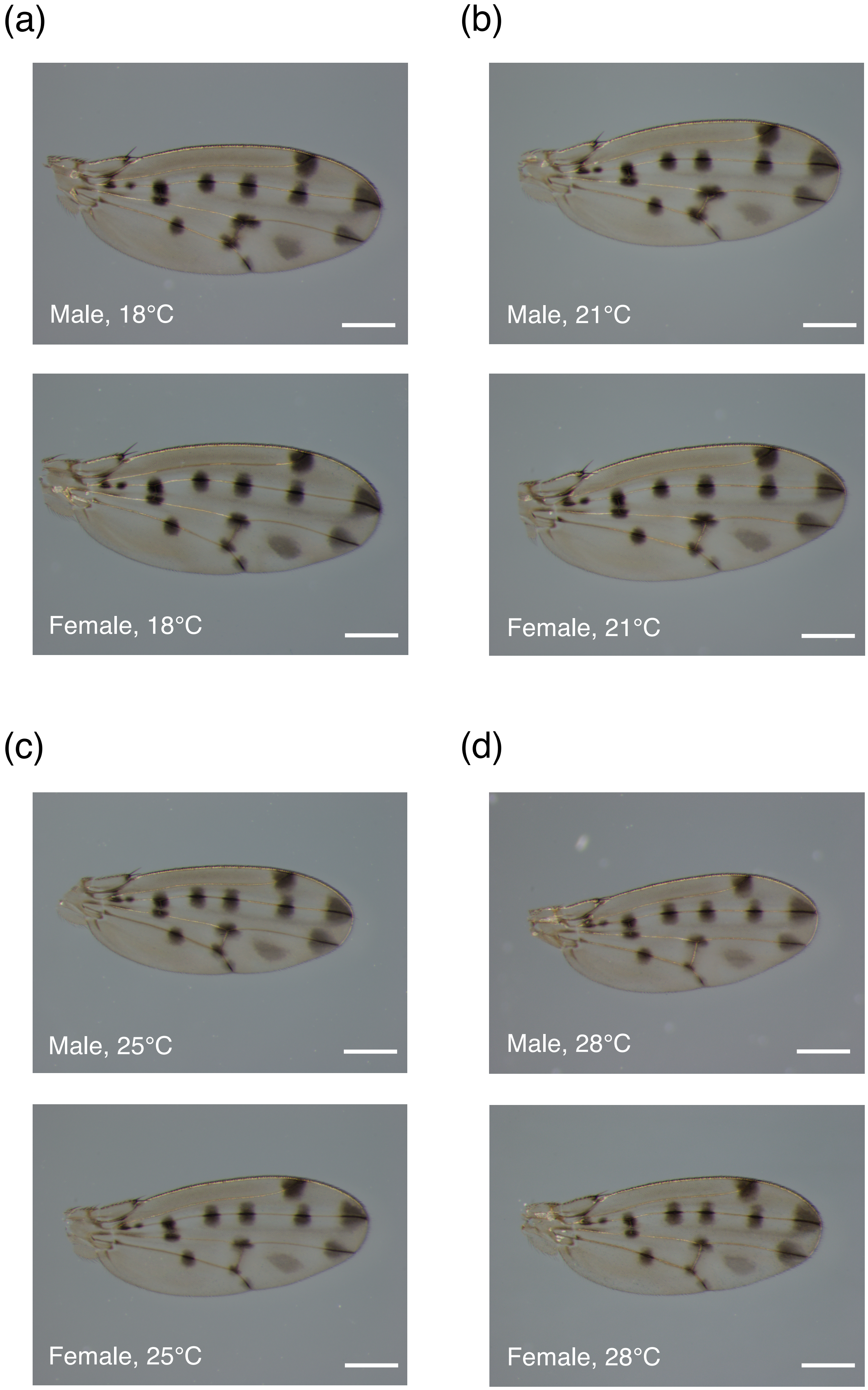
(a)
Male, 18°C
Female, 18°C
(b)
Male, 21°C
Female, 21°C
(c)
Male, 25°C
Female, 25°C
(d)
Male, 28°C
Female, 28°C
